# Index-Catalogue of the Library of the Surgeon-General’s Office

**Published:** 1885-10

**Authors:** 


### INDEX-CATALOGUE OF THE LIBRARY OF THE SURGEON-GENERAL'S OFFICE. VOL. VI. HEASTIE-INSFELDT.

---

This work is due chiefly to the zeal of Dr. Billings. The neatness and perfection of this addition to the previous five volumes indicate a like painstaking in the preparation of this valuable collection of the names of authors and the subjects treated of, including 7,900 authors-titles, representing 2,543 volumes, and 7,250 pamphlets. It also includes 14,590 subject-titles of separate books and pamphlets, and 35,290 titles of articles in periodicals—all this in one volume. Apart from affording a useful guide to those desiring information upon special subjects, this work enables those writers who are fond of embellishing their productions with long lists of references to do so without the labor of reading the books, yet we would remark, in passing, that this is a custom more honored in the breach than by the observance, both as to honesty and utility. It may not be out of place to state, that, by depositing an amount of money corresponding more or less to double the value of the books or pamphlets, anything contained in this library will be furnished for temporary use by those desiring to consult authorities.

Hygiene occupies the most space of any single heading in this volume, filling 116 pages; hernia fills 85; insanity, 83, while insane takes up 73, which, belonging to the same subject, gives 156 pages to this department, thus overshadowing by forty pages the list of works on hygiene. It will doubtless surprise those who are impressed with the nothingness of homœopathy to learn that the records of this shade of a shadow fill up 23 of these double column pages. And though a recent writer has belabored himself to convince us that the term hydrophobia has been improperly applied to various other affections, thirty pages are required to give the titles of treatises upon this disease.

Infants occupy 32 pages ,and, *mirabile dictu*! 8 pages are filled with the list of works on infanticide, that damnable practice of our day and generation. Impositions only take up half a page, which indicates that the one thousandth part has not been told, or at least recorded in books. What might seem as dry *disjecta membra* collected together in this grand index-catalogue of the library connected with the Medical Department of the United States army, becomes highly attractive in view of the light cast upon the progressive steps of science and arts by the titles of works at different periods of investigations, and it is certainly a most creditable and useful work.

### ITEMS.

DR. J. M. HEAD, of Zebulon, has removed to Barnesville.

THE session of 1885-86 of Jefferson Medical College opened with over two hundred students.

DR. S. H. GRAY, of Barnesville, member of the Medical Association of Georgia since 1871, died at his home August 31st.

DR. ROBERT BATTEY, of Rome, has resigned the Presidency of the Section of Gynecology in the International Medical Congress.

DR. MARK H. O'DANIEL, after spending quite awhile in the mountains of North Georgia, has returned to his home at Milledgeville.

DR. F. A. STANFORD, of Columbus, member of Medical Association of Georgia since the organization in 1849, died in Marietta September 15th.

DR. T. O. POWELL, of Milledgeville, Superintendent of the Georgia Lunatic Asylum, was in the city a few days ago, and gave THE JOURNAL a pleasant call.

DR. R. H. TAYLOR, of Griffin, one of the most promising young physicians in the State, was married September 16th to Miss Annie Stewart, daughter of Judge Stewart, of Griffin.

THE editor of one of our evening papers, speaking of the great number of accidents occurring every day, says : "There have been a number of drownings, recently, with fatal effect."

THE American Rhinological Association will hold its next meeting at Lexington, Ky., October 6th. Dr. C. A. S. Sims, Secretary, St. Joseph, Mo., will furnish programmes to any who may apply.

MR. HARRY HUZZA, of this city, is attending lectures at Jefferson Medical College, Philadelphia. Harry is one of the most promising young men in Georgia, and we predict for him a brilliant future.

DR. ROBERT CAMPBELL, a prominent physician living near Augusta, a member of the Medical Association of Georgia, and a brother of Dr. Henry F. Campbell, died September 22d, of heart disease.

DANIEL'S TEXAS MEDICAL JOURNAL.—The September number of this journal is on our table. It is well filled with original matter, and is ably edited. It is one of the brightest and best of our many exchanges.

QUITE a number of Georgia doctors were in Atlanta at the laying of the corner-stone of the new capitol some weeks since. Among the number we mention Dr. J. L. Hamilton, Stone Mountain; Dr. Thompson, of Bluffton; Dr. J. C. Ryals, of Spring Hill; Dr. T. D. McKeown, of Jonesboro; and Dr. Ed. Anthony, of Griffin.

DR. A. W. CALHOUN, Professor of Diseases of the Eye, Ear and Throat, in the Atlanta Medical College, has just been elected President of the Section of Ophthalmology in the Ninth International Medical Congress.

ATLANTA MEDICAL COLLEGE.—The opening address in this institution will be delivered by Prof. J. D. Wilson, at the college at 11 o'clock a. m., October 7th. We are informed that the outlook for a large class is promising.

DR. F. A. SCRIBNER, of New York, representing Messrs. Wm. Wood & Co., medical publishers, will canvass the physicians in Georgia for Wood's Reference Hand-Book of Medicine. It is a work that every physician should have, and we predict for it a large sale. Dr. Scribner is just from California, where he took four hundred and fifty orders for this valuable work.

COCAINE IN THE TREATMENT OF VAGINISMUS.—Iwow ("Russkaja Medicina;" "Dtsch. Med.-Ztg.") reports the case of a woman, thirty-two years old, who had been married ten years, and had suffered with vaginismus all that time, in spite of every kind of treatment. She was of slender build, very anæmic, and easily thrown into nervous excitement. The external genitals were normal, as well as the uterus. The vagina was short. Only remnants were found of the hymen. A five-per-cent. vaseline ointment of cocaine was ordered to be rubbed upon the vulva before each coitus. This was late in February, and in April it could already be ascertained that she was pregnant. In May indisposition to coitus supervened, but it yielded to tincture of damiana leaves (twenty drops every two hours). The author attributes the cure of the vaginismus, not to the action of the cocaine alone, but in great measure to the moral effect of his positive assurance that it would do away with the pain.—*New York Med. Journal.*

THE MEANEST MAN ON RECORD.—The proud distinction of having the meanest man on record is claimed for Oakville, Can. His wife was ill and the doctor prescribed wine. As it was not easily found, the doctor sent some from his private stores. The woman died. When the doctor's bill came in, the broken-hearted widower lodged a complaint against the physician for selling liquor contrary to law.—*Boston Medical and Surgical Journal.*

IN BAD TASTE.—The *Columbus Medical Journal*, having failed to carry its point in a controversy with Dr. Daniel, falls back upon his new *Texas Medical Journal*, claiming that he arrogates to himself what others have spontaneously awarded him in wearing gracefully the mantle of Dr. Gaillard. The pedantic utterances of the Columbus parrot are quite in contrast with the clear notes of the soaring lark of Texas; or, to *change the figure*, the jackdaw in peacock's feathers differs strikingly from the game cock.

WELLS AND BACTERIA.—In *Science*, July 10th, we read as follows: In many towns and cities the privy-vaults and leaching cesspools of every house drain really into the sheet of groundwater. The soil arrests the coarse material, the grease and slime; but the swarming bacteria diffuse with ease, as much as the soluble chlorides and nitrates, and follow the flow wholly unobstructed. Into this same soil are sunk or driven the wells; and the water that is drawn for use is polluted in proportion to the number and proximity of the vaults and cesspools, on the one hand, and the thinness and sluggishness of the water-sheet on the other. In the worst wells in daily use the water is distinctly colored with sewage; but the most deadly water may carry only the germs of typhoid fever or of dysentery, and be otherwise sparklingly clear, and so pure as to pass unchallenged through the most searching chemical analysis.—*Medical and Surgical Reporter.*

THE INTERNATIONAL CONGRESS.—The Committee on Organization of the International Medical Congress held its special meeting in this city last week. The meetings were held in secret, and secretiveness seemed to have been the essential feature of the proceedings. Doubtless the gentlemen thought they were acting very wisely ; but they need hardly have taken so much trouble. When members, supposed to represent the interests of the profession, meet at a critical time, with mystic rites, refusing all information, except to members of their own clique, they at once simply overreach themselves.

There is but one inference as to last week's work. The committee has done a foolish thing, and it prefers to cloak its folly until it can be set forth as wisdom in the gilded rhetoric of the chosen organ.

The committee, we have every reason to believe, made no concessions which can be accepted as such. They filled the one hundred and twenty-odd vacancies with gentlemen sufficiently unknown to fame to have escaped nomination by them before, and presumably sufficiently poor in spirit to accept belated honors. —*Med. Record, Sept. 12.*

METEOROLOGICAL SUMMARY FOR THIS STATION FOR AUGUST, 1885.

| | |
|---|---|
| Highest Temperature, August 7th and 24th, . . . . . | 90.0 |
| Lowest Temperature, August 27th and 28th, . . . . . | 59.0 |
| Greatest Daily Range of Temperature, . . . . . . . . . | 18.5 |
| Least Daily Range of Temperature, . . . . . . . . . | 8.0 |
| Mean Daily Range of Temperature, . . . . . . . . . | 14 9 |
| Mean Daily Dew-point, . . . . . . . . . . . . . . | 67.0 |
| Mean Daily Relative Humidity, . . . . . . . . . . . | 73.7 |
| Prevailing Direction of Wind, . . . . . . . . | Northwest. |
| Total Movement of Wind, . . . . . . . . . . . | 5,139 Miles. |
| Highest Velocity of Wind and Direction, . | 25 Miles, N. and E. |
| Number of Foggy Days, . . . . . . . . . . . . . | None. |
| Number of Clear Days, . . . . . . . . . . . . . . | 13 |
| Number of Fair Days, . . . . . . . . . . . . . . | 13 |
| Number of Cloudy Days, . . . . . . . . . . . . . | 5 |
| Number of Days on which Rain or Snow fell, . . . . . | 11 |

